# 17β-estradiol effects on human coronaries and grafts employed in myocardial revascularization: a preliminary study

**DOI:** 10.1186/1749-8090-1-46

**Published:** 2006-12-20

**Authors:** Gianluca Polvani, Fabio Barili, Giuseppe Rossoni, Luca Dainese, Manuela Wally Ossola, Veli K Topkara, Francesco Grillo, Eleonora Penza, Elena Tremoli, Paolo Biglioli

**Affiliations:** 1 Department of Cardiovascular Surgery, University of Milan, Centro Cardiologico Monzino, Via Parea 4, 20138 Milan, Italy; 2 Department of Pharmacological Sciences, University of Milan, Via Balzaretti 9, 20133 Milan, Italy; 3 Division of Cardiothoracic Surgery, College of Physicians and Surgeon of Columbia University – New York Presbyterian Hospital, Columbia University Medical Center, Milstein Hospital Building, 7GN-435 177 Fort Washington Avenue, New York, NY 10032, USA

## Abstract

**Background:**

This study was undertaken to compare the *in vitro *effects of 17β-estradiol on human epicardial coronary arteries, resistance coronary arteries and on arterial vessels usually employed as grafts in surgical myocardial revascularization.

**Methods:**

Coronary artery rings (descending coronary artery, right coronary artery, circumflex coronary artery, first septal branch) and arterial graft rings (internal thoracic artery, gastro-epiploic artery) obtained from human heart donors with heart not suitable to cardiac transplantation were connected to force transducer for isometric force recording. Precontracted specimens with and without endothelium were exposed to increasing concentration of 17β-estradiol (3–30–300–3000 nmol/l) and to vehicle (0.1% v/v ethanol). We also evaluated the effects of 17β-estradiol on vessels before and 20 minutes after exposure to L-monomethyl-arginine and indomethacin.

**Results:**

17β-estradiol induced a significant relaxation in all precontracted vessels (mean maximum effect: 78,6% ± 8,5). This effect was not different among the different rings and was not related to the presence of endothelium. N-monomethyl-L-arginine and indomethacin did not modify 17β-estradiol relaxant effect.

**Conclusion:**

The vasodilator action of the 17β-estradiol is similar on coronary arteries, resistance coronary arteries and arterial vessels usually employed as grafts in myocardial revascularization.

## Background

The interest for 17β-estradiol as vasoactive and vasoprotective agent is raising, since it was observed that it takes effect directly on the vascular wall, improving vasodilatation and inhibiting neointimal proliferation [1-7]. New devices, such as 17β-estradiol-eluting- stents, were developed to protect revascularized heart [8], hypothesizing a new role of 17β-estradiol for tertiary prevention in coronary artery disease (CAD).

Coronary perfusion after coronary artery bypass grafting (CABG) is a complex system dependent on several factors, including gender and the type of grafts employed [9-14]. Women have smaller coronary arteries than men and it can lead to incomplete revascularization and increased risk of in-hospital mortality [10]. The type of graft employed can affect outcomes as arterial grafts permit superior long-term patency and lower mortality rate [11-14]. Moreover, blood flow distribution in cardiac wall is related to not only diastolic pressure and section area of epicardial vessels but also depends on the resistance to the blood flow determined by intramyocardial branches, i.e. first septal branch [15].

The effect of estrogen on coronary system after surgical myocardial revascularization should be evaluated considering together all vessels that permit blood circulation, including grafts, epicardial coronary arteries and resistance vessels. To date, a comprehensive evaluation of estrogenic action on coronary arteries system was not performed. This study was undertaken to compare the effect of 17β-estradiol on epicardial coronary arteries, resistance coronary vessels and arteries employed as grafts in CABG.

## Methods

We evaluated the *in vitro *effect of 17β-estradiol on human epicardial coronary arteries (anterior interventricular artery, right coronary artery, circumflex artery) resistance vessels (first septal branch) and arteries usually employed in CABG (left internal mammary artery, gastroepiploic artery).

The vessels were obtained from 11 human donors whose heart was not suitable for cardiac transplantation and was harvested for banking cryopreserved valvular homografts. All patients were female (mean age 38 ± 11 years, range 18–54 years). All women had normal coronary arteries, without macroscopic atherosclerotic process.

Coronaries were dissected within 1 hours after the removal of heart and all segments were immediately put in a modified Krebs solution (composition in mmol/l: NaCl 118.3, KCl 4.7, CaCl_2 _2.5, MgSO_4 _1.2, KH_2_PO_4 _1.2, NaHCO_3 _25, EDTA Calcium 0.026, glucose 11.1, albumin 0.1) at 4°C to be conserved. At the time of experiment, at maximum 1 hour after dissection, the vessels were cut into 3–4 mm long rings and suspended in an organ bath containing 10 ml modified Krebs solution aerated constantly with 95% O_2 _and 5% CO_2 _and maintained at 37°C. Each ring was mounted on a triangular-shaped metal hook connected to force transducer for isometric force recordings. Resting force was 2 grams [16].

**Figure 1:**
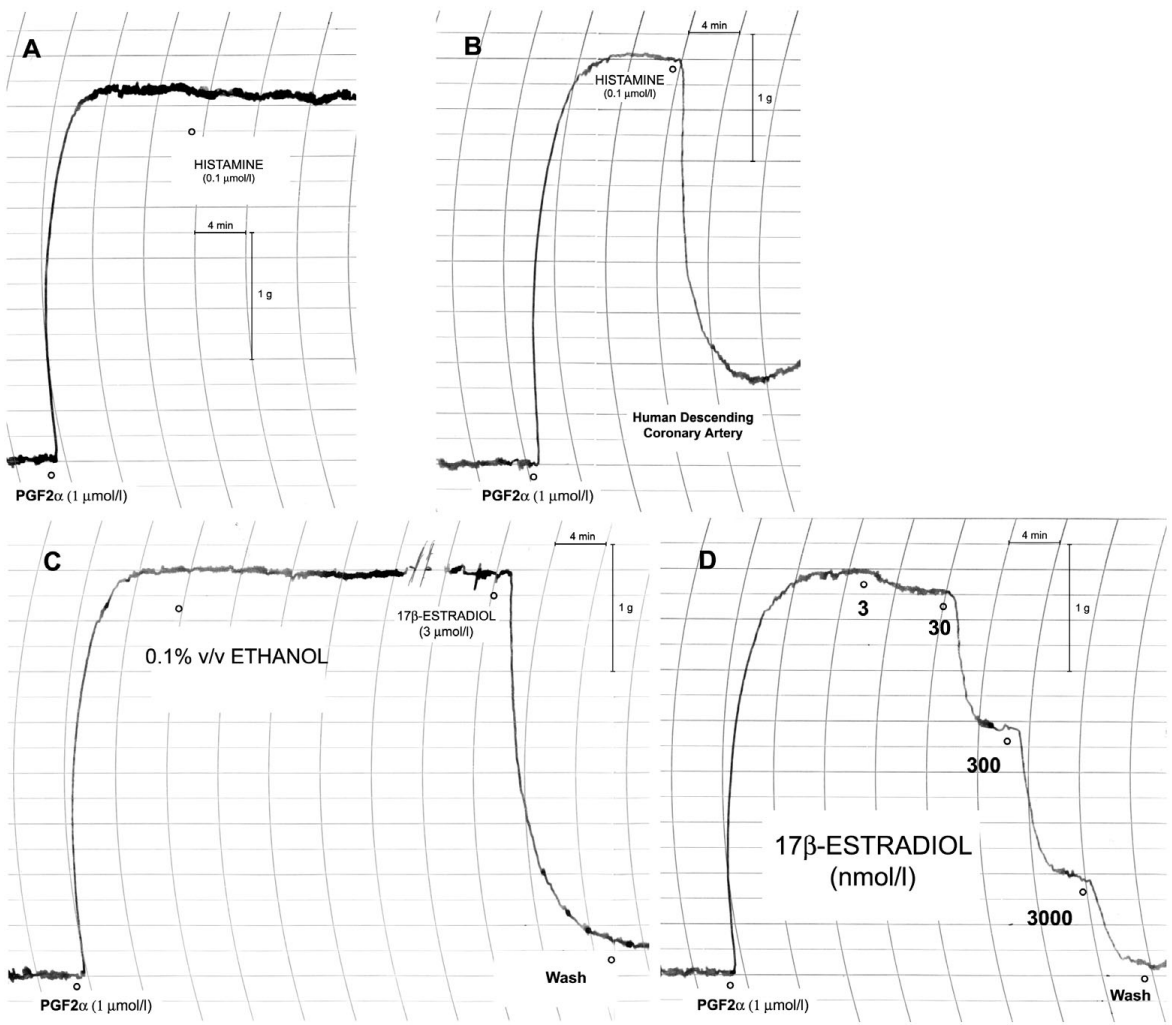
A recording showing the relaxant effect of 17β-estradiol on human female coronary arteries. In panel A, histamine had no effects on a denuded ring. In panel B, endothelium was preserved and histamine had a relaxant effect. In panel C, a ring was precontracted with PGF_2α _and exposed to the solvent (ethanol), without vasorelaxation. Adding 3 μM of 17β-estradiol to the organ bath with ethanol, the force transducer recorded the maximum decrease in force within 10 minutes. Panel D shows the dose-dependent relaxation of a precontracted ring exposed to increasing concentrations of 17β-estradiol (3–30–300–3000 nmol/l).

**Figure 2:**
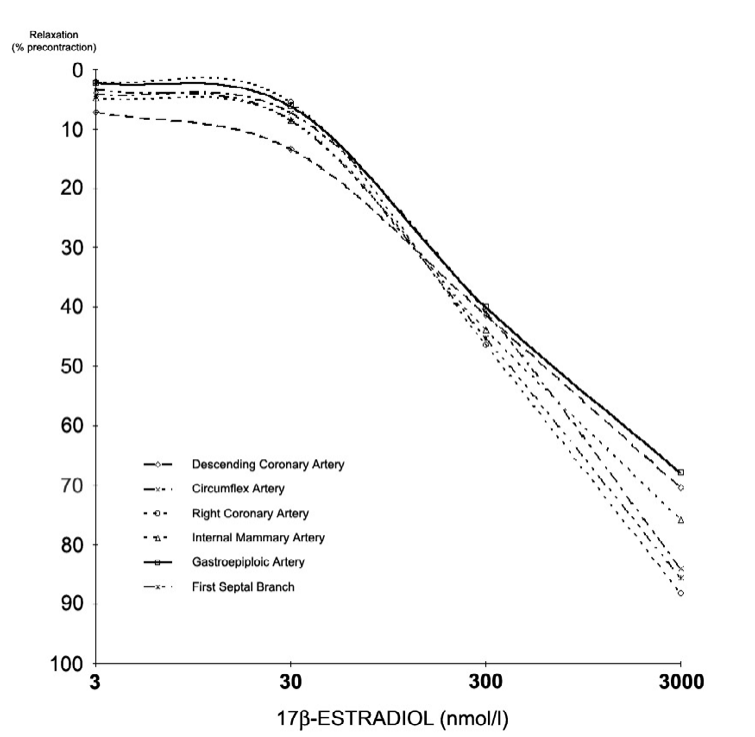
The effects of 17β-estradiol on different vessels with and without endothelium. The effect of 17β-estradiol on vessels is expressed as the percentage relaxation of the maximum contraction induced by PGF_2α_. The relaxant effect of 17β-estradiol at each dose was similar in all groups (n = 22 for each group, p > 0.05 by repeated-measures analysis of variance). No intra-group significant difference was found between the same vessels with and without endothelium (data not shown). 17β-estradiol has a similar vasoactive effect on both epicardial coronaries and septal branch and arteries usually used as graft in myocardial revascularization at each concentration.

Each single experiment was conducted on two rings from the same vessel. In one ring endothelium was mechanically removed with a wooden applicator (Group 1) in order to simulate an atheromasic vessel. The ring was precontracted with prostaglandin F_2α _(PGF_2α_, 1 μmol/l), then histamine (0.1 μmol/l) was added and the absence of vasodilative effect confirmed the complete removal of the endothelium (Figure 1-A). In the other ring from the same vessel, endothelium was preserved (Group 2). It was precontracted with prostaglandin F_2α _(PGF_2α_, 1 μmol/l) and exposed to the same concentration of histamine (0.1 μmol/l) with subsequent complete vasodilatation (Figure 2-B).

The rings (Group1 and Group2) were washed out and precontracted with prostaglandin F_2α _(PGF_2α_, 1 μmol/l). After the contraction plateau was reached (about 10 minutes), rings were exposed to the vehicle (0.1% v/v ethanol, Figure 1-C). The bath solution was replaced, the rings were precontracted again with prostaglandin F_2α _(PGF_2α_, 1 μmol/l) and, at the contraction plateau, they were exposed to increasing concentrations of 17β-estradiol (3–30–300–3000 nmol/l, Figure 1D).

At the end of the experiment, the rings were washed out and we evaluated the effects of increasing concentrations of 17β-estradiol after the exposition to L-monomethyl-arginine (L-NMMA, 0.1 mmol/l) and indomethacin (10 μmol/l), L-NMMA is a non-specific inhibitor of nitric oxide synthase (NOS) that permits to evaluate the role of nitric oxide (NO) in vasoactive action of estrogen. Indomethacin is a cyclo-oxygenase inhibitor that blocks endothelial synthesis of prostacyclin. The rings were pretreated with both L-NMMA and indomethacin together for 20 minutes and precontracted with prostaglandin F_2α _(PGF_2α_, 1 μmol/l). After the contraction plateau was reached (about 10 minutes), rings were exposed to increasing concentrations of 17β-estradiol (3–30–300–3000 nmol/l).

This study had the approval of our Institutional Ethics Committee.

### Statistical analysis

The effect of 17β-estradiol on vessels was expressed as the percentage relaxation of the maximum contraction induced by PGF_2α_. Continuous variables were expressed as mean ± standard deviation of the mean (SD). Differences between two groups were evaluated using Student's t-test. Repeated-measures analysis of variance (ANOVA) was used to compare more than two means. If statistically significant, Student's paired *t *test was then performed, with Bonferroni's method used to correct for multiple comparisons. A *p *value of less than 0.05 was considered statistically significant. Statistical analyses were performed using SPSS 13.0 software (SPSS, Inc, Chicago, IL).

## Results

17β-estradiol induced significant relaxation of precontracted coronary artery segments and vessels employed in CABG (compared with vehicle solvent, p < 0.05, data not shown). This vascular response to 17β-estradiol was concentration-dependent with a maximum effect at 3 μmol/l-dose (mean maximum effect: 78.6% ± 8.5%).

There were no significant differences in vasorelaxation between different types of vessels (p > 0.05, n = 22 in each group; Figure 2). It suggests that estrogen effect on vascular system is not dependent on vascular district and on segment's size.

**Figure 3:**
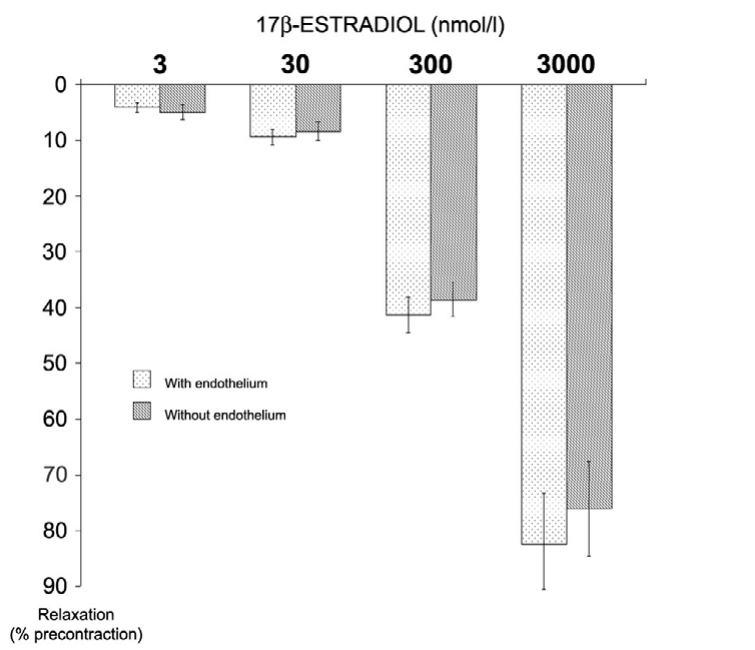
The effect of increasing concentrations (3–30–300–3000 nmol/l) of 17β-estradiol on vessels with and without endothelium. The relaxation is expressed as the percentage of the maximum effect obtained with PGF_2α_. The peak tension with PGF_2α _was 3.2 ± 1.3 for intact vessels and 3.4 ± 1.0 for denuded vessels. There were no significant differences in vascular response to estrogen in groups with or without endothelium at each estrogen concentration (n = 66 for group with endothelium, n = 66 for group without endothelium, p > 0.05).

**Figure 4:**
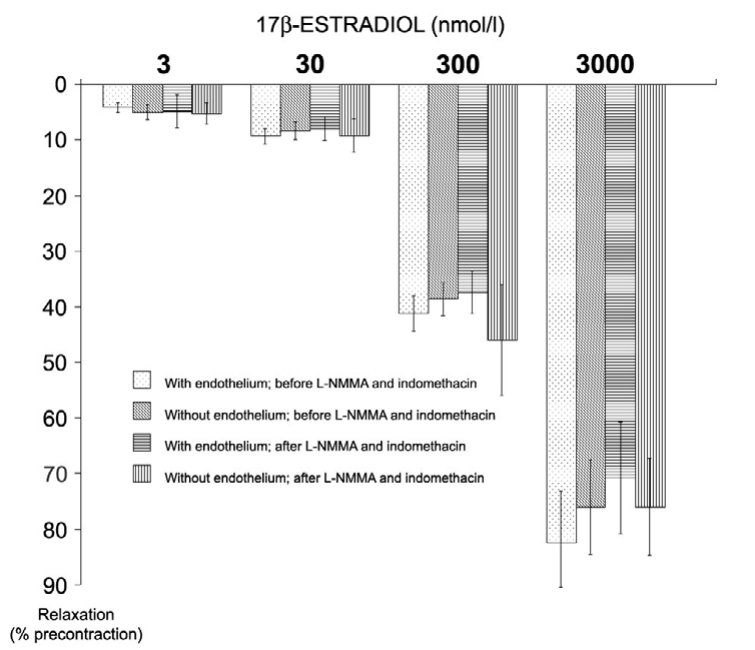
The effect of L-NMMA (0.1 mmol/l) and indomethacin (10 μmol/l) on 17β-estradiol vasorelaxation. The relaxation is expressed as the percentage of the maximum effect obtained with PGF_2α_. The peak tension with PGF_2α _was 3.3 ± 0.9 for experiment before exposure to L-NMMA and indomethacin and 3.1 ± 1.2 for the experiments after exposure to L-NMMA and indomethacin. We did not find significant differences among groups at each 17β-estradiol concentration (n = 132 in each group, p > 0.05). No intra-group significant difference was found between different vessels and between vessels with and without endothelium (p > 0.05, data not shown).

The relaxant effect of estrogen was similar in groups with and without endothelium (p > 0.05, n = 66 in each group, Figure 3), suggesting an endothelium-independent mechanism of action. L-NMMA and indomethacin did not significantly inhibit the relaxation produced by increasing concentrations of 17β-estradiol (Figure 4), excluding a role of nitric oxide and prostacyclin on estrogen-dependent relaxation.

## Discussion

The relationship between 17β-estradiol and heart has been widely evaluated in the last decade, since it was observed that the risk of coronary artery disease significantly increases in women after menopause [17]. Several clinical studies focused on the protective role of postmenopausal HRT with contrasting results that leave the debate opened [18,19]. We shifted the attention on tertiary prevention of CAD to understand the vasoactive effects of 17β-estradiol on all conduits of the revascularized heart.

Our main question regarded the eventual diverse effects of estrogen on resistance vessel, epicardial vessels and arteries commonly employed as graft in CABG. Several studies evaluated only epicardial vessels [16,20-23], without considering the importance of resistance vessels on heart perfusion. Moreover, LIMA graft was found responsible to estrogen but no comparison with coronaries was performed [24]. This study demonstrated a global acute vasorelaxant response of all vessels to 17β-estradiol which ameliorates all the complex physiology of blood flow in heart and arterial grafts. Hence, estrogen can acutely increase myocardial perfusion in women after coronary artery bypass grafting, acting through both a vasodilatation of coronary epicardial vessels and grafts and a decrease in resistance offered by resistance vessels.

The estrogenic vasoactive effect was found similar on normal and endothelium-deprived segments, confirming previous data on epicardial vessels [16,22,23]. Impaired-endothelium is characteristic of atherosclerotic coronaries and diseased arterial grafts. Surgical maneuvers are demonstrated to impair graft's endothelium and coronaries at incision site, worsening the endothelial function and leading to the well-known graft disease. The endothelium-independent vasorelaxation can be helpful in preventing perioperative vasoconstriction due to impaired endothelium and arterial graft spasm [23,24]. Moreover, estrogens accelerate endothelial cells growth, increasing local expression of vascular endothelial growth factors and inhibiting endothelial cells apoptosis [25]. This estrogen-related rapid reendothelialization, as well as vasorelaxation and inhibition of neointimal proliferation, led to the development of new estrogen-eluting stents [8] and can also represent protective effects for surgical revascularized heart. It could be useful especially in female sex, in which perioperative and postoperative complications are increased by an unfavorable anatomy [9,10].

The vasorelaxant mechanisms of 17β-estradiol on vascular conduits are far to be completely clarified [26]. New data about non-genomic mechanisms of action lead to consider 17β-estradiol also as an acute and mid-term vasodilator. 17β-estradiol both stimulates endothelial NO production in a non-genomic manner and has vasorelaxant effects on impaired vessels acting on the muscular layer through an endothelium-independent mechanism. Smooth muscular cells respond to estrogens stimulating myocyte NO-synthesis or through similar Ca-antagonist mechanisms [16,23,27,28]. Our study confirms the similar Ca-antagonist mechanism, as L-NMMA (N-monomethyl-L-arginine) does not change vascular response, even if we did not evaluate the myocyte NO-synthesis.

### Limitations of the study

This study was performed on *in vitro *specimens and the concomitant *in vivo *effects could not be evaluated. By its nature, it did not considered chronic estrogenic effects that can be related to different mechanisms, such as genomic induction. Moreover, we focused the attention on endothelium-independent mechanisms similar to Ca-antagonist, as they are responsible of vasodilatation on both normal and impaired vessels, which are characteristic of revascularized heart.

## Conclusion

This study demonstrated that 17β-estradiol has a similar relaxant effect on human female coronary arteries (epicardial capacitance arteries and resistance vessels) and arteries used as graft in CABG. Acute estrogenic administration can have vasorelaxant effect on all female revascularized heart, thus protecting coronaries and grafts and favoring reendothelialization.

## List of abbreviations

ANOVA analysis of variance

CABG coronary artery bypass grafting

CAD coronary artery disease

HRT hormone replacement therapy

L-NMMA L-monomethyl-arginine

LIMA left internal mammary artery

NO nitric oxide

NOS nitric oxide synthase

PGF_2α_, prostaglandin F_2α_

SD standard deviation of the mean

## Competing interests

The author(s) declare that they have no competing interests.

## Authors' contributions

**GP **conceived of the study, and participated in its design and helped to draft the manuscript. **FB **participated in the study's design and coordination, performed the statistical analysis and drafted the manuscript. **LD **harvested hearts form human donors and harvested all specimens for the study. **EP FG and ET **participated in the study's design and helped to perform the statistical analysis. **VKT **participated in the study's design helped to draft the manuscript and edited it. **GR **and **MVO **carried out the "in vitro" experiments. **PB **coordinated the study and participated in its design.

All authors read and approved the final manuscript.
